# Women's empowerment and intimate partner violence among pregnant women in Northern Ghana: evidence from coarsened exact matching and double-selection LASSO analyses

**DOI:** 10.3389/fgwh.2026.1905930

**Published:** 2026-10-01

**Authors:** Joseph Lasong, Yula Salifu, Yaa Nyarko Adjeso

**Affiliations:** 1 Department of Population, Family and Reproductive Health, University for Development Studies, Tamale, Ghana; 2 Department of Population, Family and Reproductive Health, University of Ghana, Accra, Ghana

**Keywords:** coarsened exact matching, Double-Selection LASSO, economic empowerment, Ghana, household decision-making, intimate partner violence, reproductive autonomy, women's empowerment

## Abstract

**Background:**

Intimate partner violence (IPV) is a major public health problem in sub-Saharan Africa. Its relationship with women's empowerment is theoretically contested, and most studies collapse empowerment into a single composite score, which may mask dimension-specific associations. We examined three empowerment dimensions including reproductive autonomy, household decision-making, and economic empowerment against physical, sexual, and emotional IPV among pregnant women in Northern Ghana.

**Methods:**

We surveyed 260 pregnant women attending antenatal care (April–May 2023). Coarsened Exact Matching (CEM) balanced high- and low-empowerment women on age, education, and number of children, retaining *n* = 246 for the pre-specified primary analysis. Double-Selection LASSO (DSL) logistic regression, applied to the full analytical sample (*n* = 260), served as a complementary sensitivity analysis. Balance after matching was assessed by standardised mean differences (SMDs) and variance ratios.

**Results:**

Any-IPV prevalence was 59.6%. Two associations showed convergent evidence across both Methods: (1) reproductive autonomy was protective against sexual IPV (CEM OR = 0.34, 95% CI 0.19–0.61; DSL OR = 0.36, 95% CI 0.20–0.63) and emotional IPV (CEM OR = 0.29, 95% CI 0.16–0.54; DSL OR = 0.36, 95% CI 0.20–0.64); and (2) household decision-making was positively associated with physical IPV in a backlash-direction pattern (CEM OR = 1.34, 95% CI 1.07–1.66, *p* = 0.009; DSL OR = 1.26, 95% CI 1.03–1.55, *p* = 0.028). Economic empowerment and the composite empowerment index were not significantly associated with any IPV outcome in either analysis.

**Conclusions:**

Reproductive autonomy showed a protective association with sexual and emotional IPV in the two different analytic approaches. Household decision-making showed a convergent positive association with physical IPV consistent with the backlash hypothesis. Composite empowerment scores obscured all dimension-specific patterns. Interventions strengthening reproductive autonomy, particularly contraceptive access and sexual-consent education, may reduce IPV vulnerability more directly than broad aggregated empowerment measures.

## Background

Intimate partner violence (IPV), defined as physical, sexual, or psychological abuse by a current or former partner, is one of the most prevalent violations of women's health and rights worldwide. The World Health Organization estimates that approximately 27% of women aged 15–49 have experienced physical or sexual partner violence in their lifetime, with prevalence rising to 33%–37% in sub-Saharan Africa, though country-level estimates vary considerably (1, 2). The health consequences are well documented and include physical injury, depression, post-traumatic stress, adverse reproductive and maternal outcomes, and elevated HIV risk (3, 4).

Considerable progress has been made in documenting how much IPV occurs; far less is known about why some women are more vulnerable than others, and specifically about how distinct dimensions of women's empowerment relate to distinct forms of violence. In Ghana, successive rounds of the Demographic and Health Survey have documented substantial and persistent IPV prevalence, with emotional violence typically the most reported subtype (5). Analysis of the 2022 Ghana Demographic and Health Survey found that a substantial minority of women still justify partner violence under certain circumstances, consistent with normative tolerance of controlling behaviour that may also depress disclosure of emotional and psychological abuse in survey settings (6). A recent study among pregnant women in Accra reported an overall IPV prevalence of 11.2%, with emotional and psychological abuse the most common subtypes (7), while research in the Northern Region found considerably higher prevalence among antenatal populations (8), highlighting the need for setting-specific evidence.

Two theoretical frameworks compete to explain how empowerment relates to IPV. Resource theory and feminist empowerment perspectives hold that greater control over resources, decisions, and reproduction increases a woman's capacity to avoid or leave dangerous relationships and to negotiate safer sex and should therefore reduce IPV risk (9–11). The male-backlash hypothesis holds the opposite: where a partner perceives a woman's growing autonomy as a threat to his authority, empowerment can provoke retaliatory violence, particularly in settings where masculine control norms are strong (12, 13). Both mechanisms plausibly operate at once, in that; empowerment changes household bargaining power, and whether that shift reduces or increases conflict depends on gender norms, economic dependence, and relationship dynamics specific to the couple and community. A single composite empowerment score cannot distinguish these opposing pathways; if one dimension is protective and another is provocative, they can cancel out in an aggregated index and produce a spurious null result (14).

Empirical evidence from the region reflects exactly this ambiguity. Cross-national Demographic and Health Survey analyses have found women's economic resources protective against IPV in some specifications (15, 16), while other multi-country analyses, drawing on data from 18 sub-Saharan African countries including Ghana, have found that women's household decision-making capacity is associated with higher, not lower, odds of IPV, consistent with the backlash hypothesis (17, 18). A recent pooled analysis using the DHS-derived Survey-based Women's Empowerment Index (SWPER) across sub-Saharan Africa, including Ghana, found that all three empowerment sub-indices were significantly associated with IPV, but not uniformly in the same direction (19), directly supporting the case for dimension-specific rather than composite measurement. None of these multi-country analyses applied matching or penalised confounder-selection methods within a Ghanaian sample specifically, both of which are suited to observational IPV data where the number of IPV events relative to candidate covariates is often modest.

## Novelty and contributions

This study makes three distinct contributions to literature, which we state explicitly. First, it is, to our knowledge, the first study of pregnant women in Northern Ghana to disaggregate women's empowerment into three conceptually distinct dimensions including reproductive autonomy, household decision-making, and economic empowerment, and to examine each against three distinct IPV subtypes, permitting dimension-specific and subtype-specific conclusions that composite-index studies cannot yield. Second, we apply two complementary confounder-adjustment methods in parallel, that is; Coarsened Exact Matching (CEM), which balance observed confounders by design, and Double-Selection LASSO (DSL), which selects confounders in a data-driven, outcome-specific manner. The rationale for selecting these two methods, including their respective strengths and limitations, is detailed in the Statistical Analysis section. Third, it provides setting-specific evidence on the empowerment–IPV relationship during pregnancy in a region where such evidence is sparse, with direct implications for antenatal screening and reproductive-autonomy programming.

## Conceptual framework

This study is guided by the ecological framework for understanding violence (16, 20), in which individual-, relationship-, community-, and societal-level factors jointly shape IPV risk. Reproductive autonomy and economic empowerment sit primarily at the individual level; household decision-making operates at the relationship level, mediated by the partner's response to a shifting balance of authority. The backlash hypothesis is incorporated explicitly as a competing pathway at the relationship level. This framework is the basis for analysing each empowerment dimension separately rather than relying on a composite score.

## Methods

### Design, setting, and objectives

This cross-sectional study was conducted between April and May 2023 among pregnant women in a current partnership attending antenatal care in the Tamale Metropolis, Northern Region, Ghana (population 233,252; 50.3% female; 81% urban) (21). Three facilities were sampled: Tamale Teaching Hospital, selected purposively for its referral role and large client base, and two secondary hospitals randomly selected from four eligible facilities. Details can be found in Salifu et al. (8).

The study had three objectives: (1) estimate the prevalence of physical, sexual, and emotional IPV; (2) identify dimension-specific empowerment correlates of each IPV subtype using CEM-adjusted logistic regression as the primary analysis and DSL logistic regression as a sensitivity analysis; and (3) evaluate model discrimination for the single empowerment–IPV combination pre-specified as most likely to be robust across both methods.

### Participants and sample size

Eligible women were pregnant, in a partnership of at least 12 months, aged 18 or older, resident in the Tamale Metropolis for at least six months, and provided written informed consent. Women with high-risk pregnancies (placenta praevia, severe pre-eclampsia, multiple gestation) were excluded, as these conditions are associated with more frequent clinical contact and psychological distress that could independently affect IPV disclosure.

Sample size was calculated using the Cochran (22) formula for a single proportion (see equation 1):

$$n=Z\alpha^{2}\times p(1-p)/e^{2}$$ (1)

with Z*α* = 1.96, *p* = 0.112 (pregnancy-specific IPV prevalence reported by Engmann et al., 2024) (7), and e = 0.05, giving a minimum requirement of 169 after a 10% allowance for non-response. Probability-proportionate-to-size allocation distributed the sample across facilities, and systematic sampling with an interval of three was applied within each facility (Kindly see details in Salifu et al., 2026) (8).

#### Sample size adequacy for CEM and DSL

With 260 enrolled women and IPV subtype counts ranging from 90 events (physical) to 152 events (emotional) against up to ten covariates, the events-per-variable (EPV) ratios ranged from approximately 9:1 to 15:1. This is at or near the conventional minimum of 10 EPV for stable logistic regression (23, 24). The physical-IPV models sit slightly below this threshold, which is one reason their confidence intervals are wider. DSL's penalised, data-driven variable selection is specifically designed to limit overfitting relative to an unpenalised model that includes every candidate covariate (25), making it suitable for settings where EPV is modest. For CEM, the matched sample of *n* = 246 retains 94.6% of the analytical sample across 12 strata. This retention rate is consistent with recommendations for CEM in moderate-sized observational studies (26).

### Data collection

Five enumerators holding Master of Public Health degrees, fluent in English and Dagbani, were trained for two days. The questionnaire was adapted from the Ghana 2022 Demographic and Health Survey instrument (5) and supplemented with items drawn from prior sub-Saharan African IPV research (6). It was pre-tested on 30 eligible women at a separate reproductive and child health unit and revised before use. Interviews (20–45 min) were conducted in English or Dagbani in private settings.

### Measures

#### Women's empowerment

Women's empowerment was scored across three dimensions, consistent with the multidimensional approach recommended by Malhotra et al. (27): reproductive autonomy (0–2: ability to refuse sex and to decide on contraceptive use; mean 0.25, SD 0.48); household decision-making (0–4: number of domains such as food, purchases, healthcare, visits in which the woman reports joint or sole decision-making authority; mean 0.99, SD 1.37); and economic empowerment (0–3: income-earning activity, access to financial resources, and control over earned income; mean 1.28, SD 0.68). The composite (0–9) was the arithmetic sum of the three dimensions, also standardised to a z-score for regression (mean 2.53, SD 1.65); a binary high-empowerment indicator used the upper tertile as threshold.

#### Intimate partner violence

IPV was assessed with items adapted from the WHO Multi-Country Study on Women's Health and Domestic Violence (28): physical IPV (ever slapped, kicked, hit, beaten, or choked by a partner), sexual IPV (ever physically forced into sex or another sexual act), and emotional IPV (ever subjected to controlling behaviour, threats, humiliation, or psychological harm by a partner). Any IPV indicates at least one subtype.

#### Covariates

Covariates, chosen from the ecological framework and prior literature, were: woman's age and education (years), number of living children, urban/rural residence, spouse's age and education, partner alcohol use, whether the woman witnessed her father beat her mother in childhood, and household wealth tertile.

### Statistical analysis

#### Rationale for method selection

CEM and DSL were selected because they address complementary threats to validity in observational IPV research:

CEM (26) is a non-parametric matching method that coarsens continuous covariates into bins and exactly matches treated and control units within strata. Its principal strength is that balance on matched covariates is achieved by design, not by functional-form assumptions in a regression model. This eliminates the risk of model misspecification driven by the result. Its principal limitation is that it can discard a substantial proportion of the sample when strata lack a counterfactual, reducing statistical power and potentially limiting generalisability. CEM was designated the primary analysis *a priori* because design-based balance is a stronger foundation for inference than model-based adjustment.

DSL (25) is a penalised regression method that selects confounders through two separate LASSO steps including one for the outcome, one for the exposure, and includes the union in the final model. Its principal strength is that it selects confounders in a data-driven, outcome-specific manner, limiting both omitted-variable bias and overfitting from a researcher-chosen covariate set applied uniformly. Its principal limitation is that it relies on the correct specification of the logistic model's functional form and does not achieve the same design-based balance as matching. DSL was designated the sensitivity analysis.

Where the two methods agree in direction and significance, we report convergent evidence. Where they disagree, we report the disagreement explicitly and interpret it as a signal for confirmatory research.

### Why data partitioning (train/test splits) was not applied

Both CEM and DSL are applied here as confounder-adjustment procedures for estimating associations, not as predictive classifiers intended for deployment on new data. Data partitioning (e.g., 70:30, 80:20, or 60:40 splits) is a validation strategy appropriate for predictive modelling, where the goal is to estimate generalisation performance on unseen data. In the present inferential context, partitioning the sample would reduce the already-modest effective sample size for each analytic method without serving the study's stated objectives. The single predictive quantity we report, that is, the AUC for the reproductive-autonomy/sexual-IPV model, is an in-sample estimate validated with DeLong confidence intervals, and we explicitly recommend external or cross-validated replication before it informs any risk-stratification application.

### Coarsened exact matching

CEM coarsens continuous covariates into bins and exactly matches women classified as high empowerment (upper tertile of composite empowerment) against the remaining women (lower empowerment) within bins of age, education, and number of children (26). Logistic regression was then fitted within the matched sample with CEM weights as analytic weights (see equation 2):

$$\mathrm{logit}[P(Y=1)]=\beta_{0}+\beta_{1}X_{1}+\beta_{2}X_{2}+\cdots +\beta_{k}X_{k}$$ (2)

where Y is the binary IPV outcome and X₁…Xₖ are the empowerment dimension of interest plus the covariate set above. Balance was assessed by standardised mean differences (SMDs) and variance ratios (VRs) for all nine covariates, before and after matching [see Supplementary Files (29)].

### Double-selection LASSO

DSL (25) selects confounders through two separate LASSO regressions: one of the outcome on candidate covariates, one of the exposure (empowerment dimension) on the same covariates, and carries forward the union of variables selected by either step into a final logistic model (see equations 3a to 3c):

$$\min\beta \;\left\{\sum {\left(Y_{i}-X_{i}\beta \right)}^{2}+\lambda_{y}\Sigma |\beta_{j}|\right\}$$ (3a, outcome model)

$$\min\gamma \left\{\sum {\left(D_{i}-X_{i}\gamma \right)}^{2}+\lambda \_d\sum |\gamma_{j}|\right\}$$ (3b, exposure model)

$$\mathrm{logit}[P(Y=1)]=\alpha D+\sum \{j\in S\}\delta_{j}X_{j}+\varepsilon$$ (3c, final model)

where D is the empowerment dimension of interest and S is the union of covariates selected by either LASSO step. This double-selection protects against omitted-confounder bias from either model alone or yields valid post-selection inference (25). DSL was fitted separately for each of the fifteen empowerment-dimension-by-IPV-subtype pairs, so the specific covariates retained differ across models; the full covariate selection results are reported in the Supplementary files. Robust (HC1) standard errors were used throughout, and all tests were two-sided at *α* = 0.05.

### Overfitting assessment

Three safeguards against overfitting are built into the analytic design. First, DSL's L1 penalty shrinks coefficients of weakly predictive covariates to zero, producing sparser models than unpenalised logistic regression (25). Second, requiring convergence of results across two structurally different methods (CEM and DSL) before treating an association as established provides a form of internal replication that would be undermined by overfitting in either method alone. Third, EPV ratios (9:1–15:1) are reported transparently, and confidence intervals for models with low EPV (physical IPV) are wide. This indicates appropriate uncertainty rather than overfitted precision. We nonetheless flag the physical-IPV models as potentially underpowered.

### Software

Analyses used R version 4.5.0 (30) with the cem package (31) for matching, glmnet and hdm (32) for DSL, base glm() for logistic regression and pROC (33) for ROC analysis.

### Ethics

Ethical approval was granted by the University for Development Studies Ethics Review Board (UDS/RB, 27/02/2023), with administrative clearance from the Northern Regional Health Directorate, the Tamale Metropolitan Health Directorate, and each participating hospital. Written informed consent was obtained from every participant. Participation was voluntary, and women could decline any question or withdraw without consequence. Referral pathways to counselling and support services were in place for disclosures of severe violence. The study followed the Declaration of Helsinki (2013 revision) and WHO's ethical and safety recommendations for domestic-violence research (34).

## Results

### Sample characteristics

Table 1 describes the full enrolled sample (*N* = 260; mean age 26.2 years, SD 5.3; mean 2.1 children, SD 1.3; 83.8% urban). IPV was common: 59.6% of women reported any IPV, driven mainly by sexual (57.3%) and emotional (58.5%) violence, with physical IPV somewhat less prevalent (34.6%).

**Table 1:** Sample characteristics and IPV prevalence (*N* = 260).

Variable	Value
Age, mean (SD)	26.2 (5.3)
Education, years, mean (SD)	2.8 (1.7)
Number of children, mean (SD)	2.1 (1.3)
Urban residence, n (%)	218 (83.8%)
Reproductive autonomy (0–2), mean (SD)	0.25 (0.48)
Household decision-making (0–4), mean (SD)	0.99 (1.37)
Economic empowerment (0–3), mean (SD)	1.28 (0.68)
Composite empowerment (0–9), mean (SD)	2.53 (1.65)
Any IPV, n (%)	155 (59.6%)
Physical IPV, n (%)	90 (34.6%)
Sexual IPV, n (%)	149 (57.3%)
Emotional IPV, n (%)	152 (58.5%)

IPV subtypes are not mutually exclusive.

### CEM balance

Table 2 shows CEM retained 246 of 260 women (94.6%) across 12 strata. 6 strata were dropped because they contained no counterfactual. The Supplementary files present standardised mean differences (SMDs) and variance ratios (VRs) for all nine covariates, before and after matching. For the three matching covariates, matching improved balance on number of children (SMD from −0.077 to +0.074; VR from 0.618 to 0.806) and modestly reduced the education imbalance (SMD from +0.213 to +0.155). For covariates not used in matching, partner alcohol use (SMD = + 0.199), witnessed father beat mother (SMD = −0.251), and residence (SMD = −0.207) remained outside the conventional |SMD| < 0.10 threshold (Austin, 2009). Overall, 4 of 9 covariates met the |SMD| < 0.10 criterion and 5 of 9 met the VR ∈ [0.80, 1.25] criterion after matching. Residual imbalance on non-matching covariates is addressed by the covariate-adjusted regression applied within the matched sample.

**Table 2:** Primary analysis: CEM-adjusted logistic regression (*n* = 246).

Empowerment dimension	IPV outcome	OR	95% CI	p
Reproductive autonomy	Physical IPV	0.7	0.38–1.28	0.248
Reproductive autonomy*	Sexual IPV	0.34	0.19–0.61	<0.001
Reproductive autonomy*	Emotional IPV	0.29	0.16–0.54	<0.001
Household decision-making*	Physical IPV	1.34	1.07–1.66	0.009
Household decision-making	Sexual IPV	1.16	0.93–1.45	0.183
Household decision-making	Emotional IPV	1.24	0.98–1.56	0.071
Economic empowerment	Physical IPV	0.93	0.55–1.57	0.790
Economic empowerment	Sexual IPV	0.98	0.59–1.61	0.926
Economic empowerment	Emotional IPV	1.12	0.66–1.88	0.674
Composite (z-score)	Physical IPV	1.28	0.96–1.72	0.094
Composite (z-score)	Sexual IPV	0.97	0.73–1.3	0.856
Composite (z-score)	Emotional IPV	1.04	0.77–1.41	0.780
High empowerment (binary)	Physical IPV	1.17	0.66–2.06	0.595
High empowerment (binary)	Sexual IPV	0.88	0.51–1.5	0.628
High empowerment (binary)	Emotional IPV	0.92	0.53–1.59	0.769

*p < 0.05. All models adjusted for age, education, number of children, residence, spouse age, spouse education, partner alcohol use, witnessed father beat mother, and wealth tertile, with CEM analytic weights.

Reproductive autonomy was significantly associated with lower odds of sexual IPV (OR = 0.34, 95% CI 0.19–0.61, *p* < 0.001) and emotional IPV (OR = 0.29, 95% CI 0.16–0.54, *p* < 0.001). Household decision-making was significantly positively associated with physical IPV (OR = 1.34, 95% CI 1.07–1.66, *p* = 0.009) but not with sexual or emotional IPV. Economic empowerment and the composite score were not significantly associated with any outcome (Table 2).

DSL corroborated the two primary findings: reproductive autonomy remained protective against sexual IPV (OR = 0.36, *p* < 0.001) and emotional IPV (OR = 0.36, *p* < 0.001), and household decision-making remained positively associated with physical IPV (OR = 1.26, *p* = 0.028). No other association achieved significance in the DSL analysis (Table 3).

**Table 3:** Sensitivity analysis — DSL logistic regression (*n* = 260).

Empowerment dimension	IPV outcome	OR	95% CI	p	Agrees with CEM?
Reproductive autonomy	Physical IPV	0.75	0.42–1.34	0.3271	Yes (both n.s.)
Reproductive autonomy*	Sexual IPV	0.36	0.2–0.63	<0.001	Yes (both sig.)
Reproductive autonomy*	Emotional IPV	0.36	0.2–0.64	<0.001	Yes (both sig.)
Household decision-making*	Physical IPV	1.26	1.03–1.55	0.0275	Yes (both sig.)
Household decision-making	Sexual IPV	1.13	0.91–1.4	0.2637	Yes (both n.s.)
Household decision-making	Emotional IPV	1.18	0.94–1.47	0.1500	Yes (both n.s.)
Economic empowerment	Physical IPV	0.83	0.51–1.35	0.4587	Yes (both n.s.)
Economic empowerment	Sexual IPV	0.9	0.56–1.46	0.6784	Yes (both n.s.)
Economic empowerment	Emotional IPV	0.89	0.55–1.45	0.6489	Yes (both n.s.)
Composite (z-score)	Physical IPV	1.19	0.9–1.57	0.2149	Yes (both n.s.)
Composite (z-score)	Sexual IPV	0.94	0.71–1.24	0.6766	Yes (both n.s.)
Composite (z-score)	Emotional IPV	0.98	0.74–1.3	0.8774	Yes (both n.s.)
High empowerment (binary)	Physical IPV	0.98	0.56–1.72	0.9347	Yes (both n.s.)
High empowerment (binary)	Sexual IPV	0.82	0.48–1.41	0.4697	Yes (both n.s.)
High empowerment (binary)	Emotional IPV	0.8	0.46–1.38	0.4157	Yes (both n.s.)

*p < 0.05. Confounder sets selected separately for each empowerment–IPV pair (Supplementary files). Robust standard errors are applied here. n.s means not significant.

## Discussion

This study examined three dimensions of women's empowerment against three IPV subtypes among pregnant women in Northern Ghana, using CEM as a pre-specified primary analysis and DSL as a complementary sensitivity analysis. Two associations showed convergent evidence across both methods.

Reproductive autonomy showed a strong protective association with sexual and emotional IPV that survived both methods. Women with greater reproductive autonomy had roughly 66% lower odds of sexual IPV and 71% lower odds of emotional IPV in the primary CEM analysis, with corroborating estimates from DSL. A plausible mechanism is that women who can refuse sex and decide on contraception retain a form of bodily self-determination that limits the coercive control underlying both sexual and emotional IPV (35). This is broadly consistent with qualitative evidence from Tanzania on how coercive control, infidelity, and forced sex are intertwined within relationships (36), with a recent pooled sub-Saharan African analysis finding that empowerment sub-indices are consistently associated with IPV (19), and with evidence from 10 West African countries that women's empowerment operates through multiple dimensions with distinct effects on different forms of violence (37). The non-significant association with physical IPV in both analyses is consistent with physical violence having different, less reproduction-specific drivers.

Household decision-making showed a convergent positive association with physical IPV across both methods. Nonetheless, the direction predicted by the male-backlash hypothesis (12, 13). This finding aligns with multi-country evidence spanning 18 sub-Saharan African countries, including Ghana, in which women's greater decision-making capacity was associated with higher IPV risk (17, 18). Practically, this argues against empowerment programming that increases women's household authority without also engaging partners on gender norms and communication (14, 38, 39). The finding that this backlash pattern is specific to physical rather than sexual or emotional IPV may reflect that physical violence is a more direct instrument of authority reassertion, whereas the coercive-control mechanisms underlying sexual and emotional IPV respond to a different dimension of empowerment (reproductive autonomy).

Economic empowerment was not significantly associated with any IPV outcome in either analysis. The composite empowerment score was likewise unrelated to any outcome. We consider this null result informative, as it directly demonstrates how aggregating dimensions with opposing associations produces a misleading zero-sum index (27).

### Comparison with previous work

Our findings both converge with and diverge from existing literature. The protective effect of reproductive autonomy is consistent with the broader evidence base linking women's bodily autonomy to reduced IPV risk (15, 16) and with the SWPER-based analysis by Donkoh et al. (19), which found the attitude-towards-violence sub-index, the SWPER component most conceptually similar to reproductive autonomy, consistently protective. The backlash-direction finding for household decision-making aligns with Ahinkorah et al. (17) but contrasts with studies in settings where women's decision-making capacity is protective, suggesting that the direction of the empowerment–IPV association is context-dependent and mediated by gender norms (18).

### Strengths and limitations

This study's principal methodological strength is applying two confounder-adjustment strategies with different bias profiles side by side. Disaggregating both empowerment and IPV into distinct dimensions and subtypes allowed the opposing associations described above to be detected.

However, several limitations qualify these conclusions. The cross-sectional design cannot establish temporal order or rule out reverse causation, that is; IPV could plausibly reduce a woman's empowerment rather than the reverse, or residual confounding by unmeasured factors such as partner personality or relationship duration. The sample is limited to 260 predominantly urban, pregnant women from one metropolis, which constrains generalisability. Self-reported IPV and empowerment are subject to social desirability and recall bias, mitigated only partly by private interviews with trained female enumerators. Empowerment dimensions were measured with short composite items rather than validated multi-item scales, which likely introduced measurement error biasing associations toward the null. CEM matching discarded 5.4% of the analytical sample, in that; the 6 dropped strata represent women for whom no adequate comparator existed. While matching improved balance on the three CEM covariates, covariates not included in the matching algorithm relied on within-matched sample regression adjustment. However, this standard two-step approach for CEM does not provide the same design-based guarantee as matching itself, and any remaining imbalance could contribute to residual confounding. EPV ratios for the physical-IPV models sit close to the conventional minimum of 10, which is one reason the physical-IPV confidence intervals are wide. Finally, the AUC reported here is an in-sample estimate. We recommend external replication before it informs any risk-prediction application.

## Conclusions

This study demonstrates that the relationship between women's empowerment and intimate partner violence is dimension- and subtype-specific. However, this pattern entirely is obscured by composite indices. Reproductive autonomy consistently protected against sexual and emotional IPV across both analytical approaches, with women reporting approximately 64%–71% lower odds. In contrast, household decision-making showed a convergent positive association with physical IPV, supporting the male-backlash hypothesis where increased domestic authority may provoke retaliatory violence in settings with rigid gender norms. Economic empowerment and aggregated empowerment scores yielded null findings, confirming that financial resources alone are insufficient without accompanying normative shifts. Programmatically, strengthening reproductive autonomy through improved contraceptive access, sexual-consent education, and legal protections against reproductive coercion, may offer some direct pathway for IPV prevention. However, initiatives that enhance household decision-making must actively engage male partners on gender equality and conflict resolution to mitigate potential backlash. Future longitudinal research is essential to establish temporal causality and validate these findings across broader Ghanaian populations to inform tailored, evidence-based interventions.

## Data Availability

The original contributions presented in the study are included in the article/Supplementary Material, further inquiries can be directed to the corresponding author.

## References

[1] World Health Organization. Violence Against Women Prevalence Estimates, 2018. Geneva: World Health Organization (2021).

[2] Aboagye RG Seidu AA Cadri A Salihu T Arthur-Holmes F Sam ST. Ending violence against women: help-seeking behaviour of women exposed to intimate partner violence in sub-saharan Africa. PLoS One. (2023) 18(10):e0291913. 10.1371/journal.pone.0291913 37796947 PMC10553277

[3] Campbell JC. Health consequences of intimate partner violence. Lancet. (2002) 359(9314):1331–6. 10.1016/S0140-6736(02)08336-8 11965295

[4] Devries KM Mak JYT García-Moreno C Petzold M Child JC Falder G. The global prevalence of intimate partner violence against women. Science. (2013) 340(6140):1527–8. 10.1126/science.1240937 23788730

[5] Ghana Statistical Service, Ghana Health Service, ICF International. Ghana Demographic and Health Survey 2022: Key Indicators Report. Accra: Ghana Statistical Service (2024).

[6] Dickson KS Ayebeng C Okyere J. Unveiling shadows: investigating women's Experience of intimate partner violence in Ghana through the lens of the 2022 demographic and health survey. PLoS One. (2024) 19(8):e0309564. 10.1371/journal.pone.0309564 39208078 PMC11361440

[7] Engmann ST Lamptey R Lawson HJ Ofori-Amankwah G. Intimate partner violence among pregnant women attending a low-resource primary care facility in Ghana. PLoS One. (2024) 19(9):e0310169. 10.1371/journal.pone.0310169 39250464 PMC11383221

[8] Salifu FM Lasong J Salifu Y. Prevalence and predictors of intimate partner violence during pregnancy in northern Ghana. Health Sci Rep. (2026) 9(4):e72351. 10.1002/hsr2.72351 42005666 PMC13083581

[9] Goode WJ. Force and violence in the family. J Marriage Fam. (1971) 33(4):624–36. 10.2307/349435

[10] Sen G. Empowerment as an Approach to Poverty. New York: UNDP (1999).

[11] Kabeer N. Gender equality and women's Empowerment: a critical analysis of the third millennium development goal. Gend Dev. (2005) 13(1):13–24. 10.1080/13552070512331332273

[12] Macmillan R Gartner R. When she brings home the bacon: labor-force participation and the risk of spousal violence against women. J Marriage Fam. (1999) 61(4):947–58. 10.2307/354015

[13] Jewkes R. Intimate partner violence: causes and prevention. Lancet. (2002) 359(9315):1423–9. 10.1016/S0140-6736(02)08357-5 11978358

[14] Salifu Y Salifu T Boakye AA Yakubu M Alhassan A Lasong J. Uneven progress: trends and social inequalities in women's Empowerment domains in Ghana from 2003 to 2022. Front Sociol. (2026) 11:1811952. 10.3389/fsoc.2026.1811952 42427804 PMC13349401

[15] Stöckl H Hassan A Ranganathan M Hatcher AM. Economic empowerment and intimate partner violence. BMC Womens Health. (2021) 21(1):241. 10.1186/s12905-021-01363-9 PMC8199805 34118922

[16] Heise L Kotsadam A. Cross-national and multilevel correlates of partner violence: an analysis of data from population-based surveys. Lancet Glob Health. (2015) 3(6):e332–40. 10.1016/S2214-109X(15)00013-3 26001577

[17] Ahinkorah BO Dickson KS Seidu AA. Women decision-making capacity and intimate partner violence among women in sub-saharan Africa. Arch Public Health. (2018) 76:5. 10.1186/s13690-018-0253-9 29423218 PMC5787915

[18] Cools S Kotsadam A. Resources and intimate partner violence in sub-saharan Africa. World Dev. (2017) 95:211–30. 10.1016/j.worlddev.2017.02.027

[19] Donkoh IE Aboagye RG Okyere J Seidu AA Ahinkorah BO Yaya S. Association between the survey-based women's Empowerment index (SWPER) and intimate partner violence in sub-saharan Africa. Reprod Health. (2024) 21:63. 10.1186/s12978-024-01755-8 38730477 PMC11088024

[20] Heise LL. Violence against women: an integrated, ecological framework. Violence Against Women. (1998) 4(3):262–90. 10.1177/1077801298004003002 12296014

[21] Ghana Statistical Service, Ghana Health Service, ICF International. Ghana Demographic and Health Survey 2014. Rockville (MD): ICF International (2015).

[22] Cochran WG. Sampling Techniques. 3rd ed New York: John Wiley & Sons (1977).

[23] Peduzzi P Concato J Kemper E Holford TR Feinstein AR. A simulation study of the number of events per variable in logistic regression analysis. J Clin Epidemiol. (1996) 49(12):1373–9. 10.1016/S0895-4356(96)00236-3 8970487

[24] Vittinghoff E McCulloch CE. Relaxing the rule of ten events per variable in logistic and cox regression. Am J Epidemiol. (2007) 165(6):710–8. 10.1093/aje/kwk052 17182981

[25] Belloni A Chernozhukov V Hansen C. High-dimensional methods and inference on structural and treatment effects. J Econ Perspect. (2014) 28(2):29–50. 10.1257/jep.28.2.29

[26] Iacus SM King G Porro G. Causal inference without balance checking: coarsened exact matching. Polit Anal. (2012) 20(1):1–24. 10.1093/pan/mpr013

[27] Malhotra A Schuler SR Boender C. Measuring women's empowerment as a Variable in International Development. Washington (DC): World Bank (2002).

[28] García-Moreno C Jansen HAFM Ellsberg M Heise L Watts CH. Prevalence of intimate partner violence: findings from the WHO multi-country study on women's Health and domestic violence. Lancet. (2006) 368(9543):1260–9. 10.1016/S0140-6736(06)69523-8 17027732

[29] Austin PC. Balance diagnostics for comparing the distribution of baseline covariates between treatment groups in propensity-score matched samples. Stat Med. (2009) 28(25):3083–107. 10.1002/sim.3697 19757444 PMC3472075

[30] R Core Team. R: a language and environment for statistical computing. Version 4.5.0. Vienna: R Foundation; 2025.

[31] Iacus SM King G Porro G. Cem: software for coarsened exact matching. J Stat Softw. (2009) 30(9):1–27. 10.18637/jss.v030.i0921666874

[32] Chernozhukov V Hansen C Spindler M. Hdm: high-dimensional metrics. R J. (2016) 8(2):185–99. 10.32614/RJ-2016-040

[33] Robin X Turck N Hainard A Tiberti N Lisacek F Sanchez JC. pROC: an open-source package for R and S+. BMC Bioinformatics. (2011) 12:77. 10.1186/1471-2105-12-77 21414208 PMC3068975

[34] World Health Organization. Putting Women First: Ethical and Safety Recommendations for Research on Domestic Violence Against Women. Geneva: World Health Organization (2001).

[35] Salifu Y Yakubu M Lasong J Alhassan A Isaac Gunu A Ogum D. Sexual autonomy among married young women in Ghana: a multivariable modified poisson and multivariate poisson decomposition analysis of a large population-based survey. Crit Public Health. (2026) 36(1):2673256. 10.1080/09581596.2026.2673256

[36] Lary H Maman S Katebalila M McCauley A Mbwambo J. Exploring the association between HIV and violence. Int Fam Plan Perspect. (2004) 30(4):200–6. 10.1363/3020004 15590386

[37] Ewerling F Lynch JW Victora CG Van Eerdewijk A Tyszler M Barros AJD. The SWPER index for women's Empowerment in Africa: development and validation of an index based on survey data. Lancet Glob Health. (2017) 5(9):e916–23. 10.1016/S2214-109X(17)30292-9 28755895 PMC5554795

[38] Dworkin SL Dunbar MS Krishnan S Hatcher AM Sawires S. Uncovering tensions and capitalizing on synergies in HIV/AIDS and antiviolence programs. Am J Public Health. (2011) 101(6):995–1003. 10.2105/AJPH.2009.191106 21164091 PMC3093292

[39] Jewkes R Flood M Lang J. From work with men and boys to changes of social norms and reduction of inequities in gender relations. Lancet. (2015) 385(9977):1580–9. 10.1016/S0140-6736(14)61683-4 25467578

