# Supplementary material for: Women's empowerment and intimate partner violence among pregnant women in Northern Ghana: evidence from coarsened exact matching and double-selection LASSO analyses
Source: Front Glob Womens Health. 2026 Oct 1;7:1905930. doi: 10.3389/fgwh.2026.1905930 (PMC13669198; doi:10.3389/fgwh.2026.1905930)
Supplement: Supplementary file 2 [file Table2.docx]

# Supplementary Files

## IPV by Empowerment Tertile

Supplementary Table 1 shows a non-monotonic pattern that anticipates the composite-index problem discussed later: the medium composite-empowerment tertile had the highest any-IPV prevalence (66.3%), not the low tertile (56.3%) or the high tertile (55.8%). Physical IPV rose steadily across tertiles (28.7% → 34.9% → 39.5%), while sexual and emotional IPV peaked in the medium tertile before falling in the high tertile. A single composite score summarising three dimensions with different, sometimes opposed, associations cannot be expected to trend cleanly, and it does not here.

Supplementary **Table 1.** IPV prevalence by composite women's empowerment tertile (N = 260)

| **Tertile** | **N** | **Any IPV** | **Physical** | **Sexual** | **Emotional** |
| --- | --- | --- | --- | --- | --- |
| Low | 87 | 56.3% | 28.7% | 54.0% | 56.3% |
| Medium | 86 | 66.3% | 34.9% | 64.0% | 62.8% |
| High | 86 | 55.8% | 39.5% | 53.5% | 55.8% |

## Correlations Among Dimensions and Outcomes

Supplementary Table 2 shows the three IPV subtypes were themselves highly intercorrelated (sexual–emotional r = 0.94; physical–emotional r = 0.60; physical–sexual r = 0.53), which is why discrimination was formally tested for only one representative outcome (see Methods). Among empowerment dimensions, household decision-making dominated the composite (r = 0.85 with the composite score), while reproductive autonomy (r = 0.46) and economic empowerment (r = 0.40) contributed less. Critically, the dimensions correlated with IPV in opposite directions: reproductive autonomy was negatively correlated with sexual (r = −0.21) and emotional (r = −0.21) IPV, while household decision-making was positively correlated with physical (r = 0.22) and emotional (r = 0.15) IPV. The composite score's correlation with all three IPV subtypes was close to zero (r = 0.11, 0.01, 0.03). We interpret this uniformly, here and wherever the composite is discussed later in this paper, as arithmetic cancellation of the oppositely signed dimension-level associations, not as evidence that empowerment in general is unrelated to IPV.

Supplementary **Table 2.** Pearson correlations: empowerment dimensions and IPV subtypes (N = 260)

| **Variable** | **Repro. autonomy** | **HH decision** | **Econ. empower.** | **Composite** | **Physical IPV** | **Sexual IPV** | **Emotional IPV** |
| --- | --- | --- | --- | --- | --- | --- | --- |
| Repro. autonomy | 1.00 | 0.14 | 0.11 | 0.46 | −0.07 | −0.21 | −0.21 |
| HH decision-making |  | 1.00 | 0.06 | 0.85 | 0.22 | 0.12 | 0.15 |
| Econ. empowerment |  |  | 1.00 | 0.40 | −0.14 | −0.07 | −0.09 |
| Composite |  |  |  | 1.00 | 0.11 | 0.01 | 0.03 |
| Physical IPV |  |  |  |  | 1.00 | 0.53 | 0.60 |
| Sexual IPV |  |  |  |  |  | 1.00 | 0.94 |
| Emotional IPV |  |  |  |  |  |  | 1.00 |

*HH = household. Negative values for reproductive autonomy indicate a protective pattern; positive values for household decision-making indicate a backlash-consistent pattern.*

## Supplementary Table 3. Covariates selected by LASSO for each empowerment–IPV pair (n = 260)

| Covariate | RA-Ph | RA-Sx | RA-Em | HH-Ph | HH-Sx | HH-Em | EE-Ph | EE-Sx | EE-Em | Comp-Ph | Comp-Sx | Comp-Em | HE-Ph | HE-Sx | HE-Em |
| --- | --- | --- | --- | --- | --- | --- | --- | --- | --- | --- | --- | --- | --- | --- | --- |
| Age | — | Y | Y | D | Y+D | Y+D | D | Y+D | Y+D | D | Y+D | Y+D | D | Y+D | Y+D |
| Education | Y | Y | Y | Y | Y | Y | Y+D | Y+D | Y+D | Y+D | Y+D | Y+D | Y+D | Y+D | Y+D |
| No. children | Y | Y | Y | Y | Y | Y | Y+D | Y+D | Y+D | Y+D | Y+D | Y+D | Y+D | Y+D | Y+D |
| Residence | — | — | — | D | D | D | D | D | D | D | D | D | D | D | D |
| Spouse age | Y | — | — | Y+D | D | D | Y+D | D | D | Y | — | — | Y | — | — |
| Spouse educ. | Y | Y | Y | Y+D | Y+D | Y+D | Y+D | Y+D | Y+D | Y+D | Y+D | Y+D | Y+D | Y+D | Y+D |
| Partner alcohol | Y | Y | Y | Y+D | Y+D | Y+D | Y+D | Y+D | Y+D | Y+D | Y+D | Y+D | Y+D | Y+D | Y+D |
| Witnessed IPV | Y | Y | Y | Y+D | Y+D | Y+D | Y+D | Y+D | Y+D | Y+D | Y+D | Y+D | Y+D | Y+D | Y+D |
| Wealth tertile | Y | — | Y | Y | — | Y | Y+D | D | Y+D | Y+D | D | Y+D | Y | — | Y |

*RA = Reproductive autonomy; HH = Household decision-making; EE = Economic empowerment; Comp = Composite (z-score); HE = High empowerment (binary). Ph = Physical IPV; Sx = Sexual IPV; Em = Emotional IPV. Y = selected by outcome LASSO; D = selected by exposure LASSO; Y+D = selected by both; — = not selected.*

## Supplementary Table 4. CEM balance diagnostics: SMDs and variance ratios

| Covariate | CEM? | Mean(T) Before | Mean(C) Before | SMD Before | VR Before | Mean(T) After | Mean(C) After | SMD After | VR After |
| --- | --- | --- | --- | --- | --- | --- | --- | --- | --- |
| Age (years) | Yes | 26.62 | 25.89 | +0.140 | 1.012 | 26.55 | 25.59 | +0.185 | 1.021 |
| Education (years) | Yes | 2.98 | 2.62 | +0.213 | 1.245 | 2.94 | 2.68 | +0.155 | 1.229 |
| Number of children | Yes | 2.07 | 2.17 | -0.077 | 0.618 | 2.07 | 1.98 | +0.074 | 0.806 |
| Residence (1=rural) | No | 1.79 | 1.87 | -0.232 | 1.517 | 1.79 | 1.87 | -0.207 | 1.452 |
| Spouse age (years) | No | 33.83 | 33.24 | +0.084 | 0.755 | 33.56 | 32.96 | +0.087 | 0.674 |
| Spouse education | No | 3.25 | 3.4 | -0.078 | 1.000 | 3.25 | 3.41 | -0.082 | 1.000 |
| Partner alcohol use | No | 0.15 | 0.09 | +0.193 | 1.596 | 0.15 | 0.09 | +0.199 | 1.616 |
| Witnessed father beat mother | No | 1.81 | 1.89 | -0.249 | 1.646 | 1.8 | 1.89 | -0.251 | 1.642 |
| Wealth tertile | No | 2.03 | 1.96 | +0.081 | 1.121 | 2.02 | 1.96 | +0.074 | 1.106 |

*T = treated (high empowerment); C = control (lower empowerment). SMD = (mean_T − mean_C) / pooled SD. VR = var_T / var_C. Thresholds: |SMD| < 0.10, VR ∈ [0.80, 1.25] (Austin, 2009). After matching: 4/9 covariates meet |SMD| < 0.10; 5/9 meet VR criterion.*

## Full Covariate-Adjusted Models for Reproductive Autonomy

Reproductive autonomy was the only dimension significant in both the primary and sensitivity analyses for more than one outcome, which is why it is reported in full in Supplementary Tables 5a and 5b, both fitted on the CEM-matched sample. Model discrimination (AUC) was evaluated only for Model A (sexual IPV); Model B (emotional IPV) is reported here for its covariate-adjusted odds ratios only.

In Model A, reproductive autonomy remained the strongest correlate of sexual IPV (OR = 0.31, p < 0.001). Witnessing a father beat the mother in childhood was associated with lower odds of sexual IPV (OR = 0.37, p = 0.041). In Model B, reproductive autonomy again dominated (OR = 0.27, p < 0.001); number of children was positively associated with emotional IPV (OR = 1.74, p = 0.019): each additional living child was associated with 74% higher adjusted odds of emotional IPV. Witnessing paternal violence again showed a protective association (OR = 0.32, p = 0.028).

Supplementary **Table 5a.** Full model — reproductive autonomy and sexual IPV (Model A)

| **Variable** | **OR** | **95% CI** | **p** |
| --- | --- | --- | --- |
| Reproductive autonomy* | 0.31 | 0.17–0.56 | <0.001 |
| Age | 1.06 | 0.95–1.18 | 0.310 |
| Education | 0.99 | 0.74–1.32 | 0.952 |
| Number of children | 1.37 | 0.89–2.11 | 0.158 |
| Residence (rural) | 0.55 | 0.19–1.54 | 0.254 |
| Spouse age | 0.99 | 0.92–1.06 | 0.825 |
| Spouse education | 0.90 | 0.70–1.17 | 0.428 |
| Partner alcohol use | 1.30 | 0.51–3.32 | 0.583 |
| Witnessed father beat mother* | 0.37 | 0.14–0.96 | 0.041 |
| Wealth tertile | 1.51 | 0.95–2.41 | 0.082 |

** p < 0.05. AIC = 321.96. Robust sandwich standard errors.*

Supplementary **Table 5b.** Full model — reproductive autonomy and emotional IPV (Model B)

| **Variable** | **OR** | **95% CI** | **p** |
| --- | --- | --- | --- |
| Reproductive autonomy* | 0.27 | 0.14–0.50 | <0.001 |
| Age | 1.06 | 0.94–1.18 | 0.333 |
| Education | 0.99 | 0.74–1.33 | 0.936 |
| Number of children* | 1.74 | 1.10–2.77 | 0.019 |
| Residence (rural) | 0.61 | 0.21–1.75 | 0.355 |
| Spouse age | 0.99 | 0.92–1.06 | 0.771 |
| Spouse education | 0.88 | 0.67–1.14 | 0.332 |
| Partner alcohol use | 1.21 | 0.45–3.23 | 0.709 |
| Witnessed father beat mother* | 0.32 | 0.12–0.88 | 0.028 |
| Wealth tertile | 1.51 | 0.93–2.44 | 0.094 |

** p < 0.05. AIC = 310.18. Robust sandwich standard errors.*

## Model Discrimination

Discrimination was formally evaluated for the reproductive-autonomy/sexual-IPV model only: AUC = 0.712 (95% CI 0.643–0.781), significantly above the chance level of 0.5. At the Youden-optimal threshold (0.537), sensitivity was 73.6% and specificity 62.5% (Supplementary Table 6). In practical terms, of every 100 women who truly experienced sexual IPV and 100 who truly did not, this model would correctly classify roughly 74 of the first group and 63 of the second, but with enough misclassification in both directions that the model is better suited to a research or screening context, alongside clinical judgement, than to stand-alone diagnostic use. This AUC was estimated in-sample. We recommend external or cross-validated replication before it informs any individual-level risk-prediction application.

Supplementary **Table 6.** Discrimination for the pre-specified primary model

| **Model** | **AUC** | **95% CI** | **Threshold** | **Sensitivity** | **Specificity** |
| --- | --- | --- | --- | --- | --- |
| Reproductive autonomy → sexual IPV (CEM-adjusted) | 0.712 | 0.643–0.781 | 0.537 | 73.6% | 62.5% |
